# Diversification under sexual selection: the relative roles of mate preference strength and the degree of divergence in mate preferences

**DOI:** 10.1111/ele.12142

**Published:** 2013-07-01

**Authors:** Rafael L Rodríguez, Janette W Boughman, David A Gray, Eileen A Hebets, Gerlinde Höbel, Laurel B Symes

**Affiliations:** 1 Behavioral and Molecular Ecology Group, Department of Biological Sciences, University of Wisconsin–MilwaukeeMilwaukee, WI, 53201, USA; 2 Department of Zoology, Ecology, Evolutionary Biology & Behavior Program; BEACON, Michigan State UniversityEast Lansing, MI, 48824, USA; 3 Department of Biology, California State University NorthridgeNorthridge, CA, 91330, USA; 4 School of Biological Sciences, University of Nebraska LincolnLincoln, NE, 68588, USA; 5 Ecology and Evolutionary Biology, Dartmouth CollegeHanover, NH, 03755, USA

**Keywords:** Diversification, mate preference function, sexual coevolution

## Abstract

The contribution of sexual selection to diversification remains poorly understood after decades of research. This may be in part because studies have focused predominantly on the strength of sexual selection, which offers an incomplete view of selection regimes. By contrast, students of natural selection focus on environmental differences that help compare selection regimes across populations. To ask how this disparity in focus may affect the conclusions of evolutionary research, we relate the amount of diversification in mating displays to quantitative descriptions of the strength and the amount of divergence in mate preferences across a diverse set of case studies of mate choice. We find that display diversification is better explained by preference divergence rather than preference strength; the effect of the latter is more subtle, and is best revealed as an interaction with the former. Our findings cast the action of sexual selection (and selection in general) in a novel light: the strength of selection influences the rate of evolution, and how divergent selection is determines how much diversification can occur. Adopting this view will enhance tests of the relative role of natural and sexual selection in processes such as speciation.

## Introduction

Nearly a century and a half ago Darwin proposed sexual selection as an explanation for the evolution of extravagant traits that could not be expected to arise under natural selection (Darwin 1871), and three decades ago biologists started to focus on sexual selection as a powerful agent of trait elaboration and speciation (West–Eberhard 1983). Since then, much work has addressed the relative contributions of natural and sexual selection to species divergence and to the diversification of traits involved in adaptation and reproductive isolation (e.g. Coyne & Orr 2004; Ritchie 2007; Ritchie *et al*. 2007; Seddon *et al*. 2008; Arnegard *et al*. 2010; Labonne & Hendry 2010; Kraaijeveld *et al*. 2010; Maan & Seehausen 2011; Wagner *et al*. 2012). Several fundamental studies have identified important differences between natural and sexual selection: Sexual selection is stronger and more constant; in addition, in sexual selection relative attractiveness is never maximised, novelty *per se* is often advantageous and the default dynamics of Fisherian runaway selection make it widespread and self-reinforcing (Darwin 1871; Fisher 1958; West–Eberhard 1983; Hoekstra *et al*. 2001; Kingsolver *et al*. 2001; Hereford *et al*. 2004; Svensson *et al*. 2006; Prum 2010, 2012; Siepielski *et al*. 2011).

**Figure 1:**
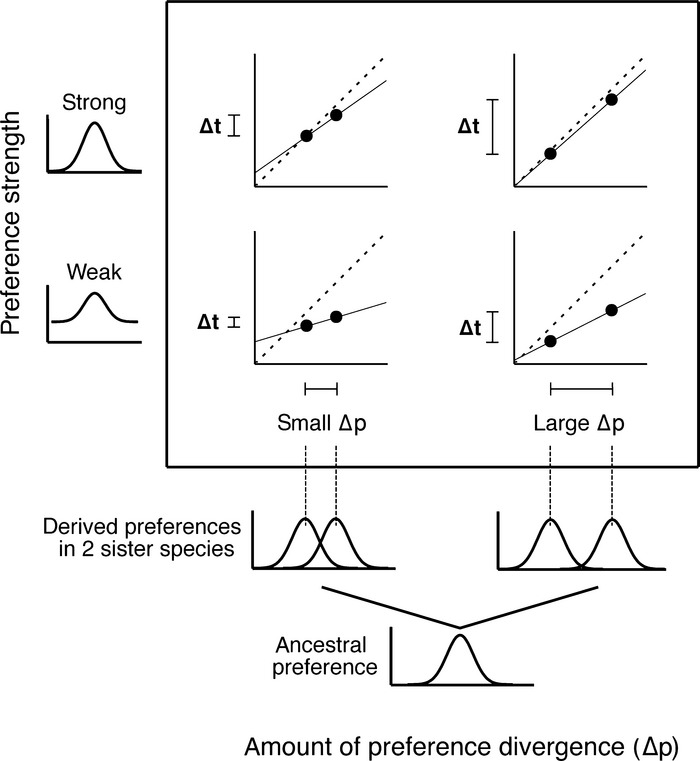
Heuristic model for the relationship between the amount of divergence in the display trait values favoured by mate preferences (labelled Δp, depicted on the *x*-axis), the strength of the mate preferences (depicted on the *y*-axis) and the resulting divergence in mating displays (labelled Δt, indicated with brackets by each panel). We show this for two sister species, indicated as the two data points in each panel. The amount of preference divergence dictates how much display divergence can occur: more divergent preferences (greater Δp) result in more divergent displays (greater Δt). Preference strength, by contrast, determines the rate of evolution (faster with stronger preferences) and the closeness of the display–preference match (closer with strong preferences). In each panel, the dotted line indicates a perfect 1 : 1 match between displays and preferences. Bottom: an ancestral and two derived preference functions, one for each sister species.

The above work has given us a good understanding of the features that enable sexual selection to generate rapid divergence and extravagance beyond naturally selected optima. A problem arises, however, when researchers take strength and rapid evolution as the key features that should characterise the action of sexual selection. This is because the key to testing hypotheses about the action of selection is to relate descriptors of selection regimes to observed patterns of divergence or diversification. In such tests, focusing on the strength of selection can be misleading because the effects of selection on diversification can only be detected in relation to the amount of divergence in the phenotypes that are favoured by selection (i.e. in fitness peaks). Consider the following heuristic model of the process of diversification of mating displays under sexual selection by mate choice (Fig. 1): Assuming sufficient genetic variation, diversification in mating displays will depend on two variables. First, the strength of selection (e.g. the strength of mate preferences) will determine how closely and how quickly display trait values come to match the fitness peaks defined by mate preferences: The display–preference match will be closer with strong preferences (Rodríguez *et al*. 2006), which are more likely to outweigh competing sources of selection such as naturally selected costs; also, the ‘equilibrium’ display–preference match may be attained more quickly with stronger preferences. Second, the amount of divergence in the display trait values that are favoured by mate preferences will determine the magnitude of the diversification that occurs in display phenotypes. The consequence is that, over evolutionary time, even weak selection can generate considerable diversification if there is a large amount of divergence in mate preferences; by contrast, stronger selection can more rapidly result in a closer display–preference match but can only account for as much divergence as exists among mate preferences (Fig. 1). The key to diversification, then, is the divergent nature of selection, rather than its strength *per se*.

It may seem obvious that the action of selection should be characterised not only by its strength but also by how divergent it is. However, this point marks a contrast in how the action of natural and sexual selection have been compared in evolutionary research. Comparative studies of speciation by ecological selection have focused on environment differences that help capture how divergent the ecological context of selection is (Boughman 2002; Schluter 2001, 2009; Rundle & Nosil 2005; Nosil 2012), whereas comparative studies of speciation by sexual selection have sought proxies for the strength of sexual selection, such as the degree of sexual dimorphism or the type of mating system (Coyne & Orr 2004; Panhuis *et al*. 2001; Ritchie 2007; Ritchie *et al*. 2007; Seddon *et al*. 2008). Explicit consideration of the extent of divergence in selection has been lacking in comparative studies of the action of sexual selection, potentially confounding comparisons of the effectiveness of ecological and sexual selection as agents of divergence and speciation.

Here, we explore the consequences of failing to capture these different aspects of the action of sexual selection. We ask how well divergence in mating displays is explained by focusing either only on the strength or on the amount of divergence in mate preferences. We use a diverse set of case studies of mate choice, drawn from our own work and from the literature when the relevant data could be obtained. The case studies feature crickets, frogs, katydids, sticklebacks, tree crickets, treehoppers, and wolf spiders (see Appendix S1 in Supporting Information). To be included in our analysis, a case study had to allow extracting quantitative information about three features related to mate choice (amount of divergence in mating display traits, amount of divergence in trait values favoured by mate preferences, and strength of mate preferences; see below) in a way that allowed comparison across traits and case studies. Furthermore, this information had to be available for at least three closely related species or populations, so that we could relate the two variables describing mate preferences to the variable describing divergence in mating display traits. In no case did we have prior knowledge of the patterns that we describe, and we did not discard contrary data, nor are we aware of other studies that meet our criteria. In fact, one of the case studies – geographic sampling of a large field cricket population for which molecular data demonstrate panmixis (Gray *et al*. 2008) – served to examine whether our analysis could generate spurious results: with panmixis there should be no divergence among sample localities, and there should be no relationship between display divergence and either preference strength or preference divergence. To our knowledge, we have only excluded two studies from the literature on population or species differences in mate preferences (Shaw & Herlihy 2000; Simmons *et al*. 2001) because they did not allow extracting comparable data for our analyses. Our case studies share the following features: The taxa involved were interesting from the perspective of research on mate choice; they deal with pair formation (as opposed to later stages of the reproductive process); and they involve ‘traditional’ sex roles whereby males compete for matings and females exercise mate choice (cf. Clutton–Brock 2007). We do not expect these commonalities to bias our contrast of the roles of the strength of sexual selection and of how divergent selection is.

**Figure 2:**
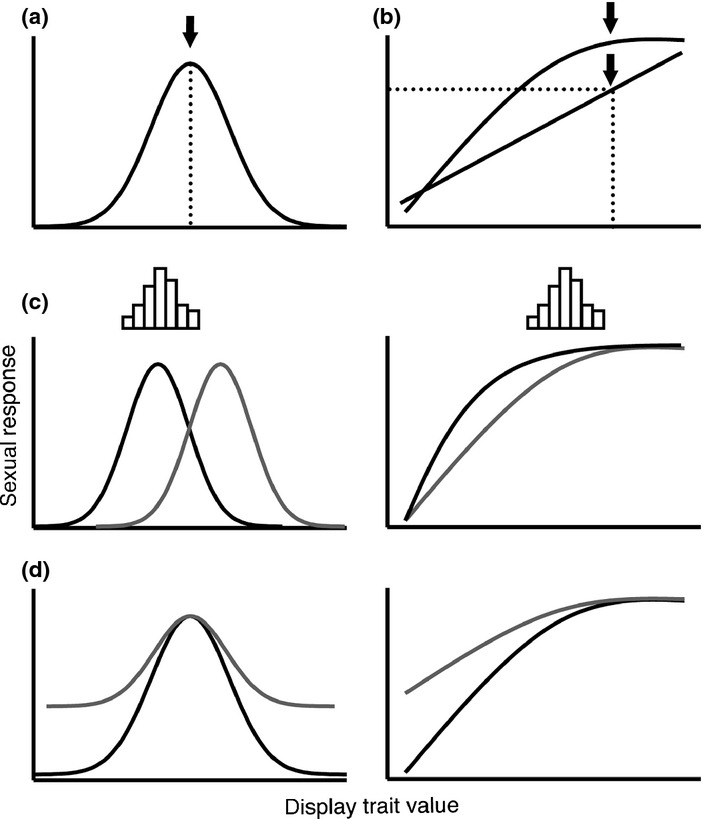
Preference functions relate variation in sexual response to variation in display traits. (a) Closed preference functions rise to peak response at the preferred display trait value (‘peak preference’; arrow) and then decline. (b) Open preference functions continue to rise or level off, although a peak may be defined (arrows) if further display investment brings diminishing returns. (c) In relation to display trait distributions (histograms), preference functions make predictions about the form of selection (see text). Here, black vs. grey functions predict stabilising vs. directional selection (closed preferences) or varying directional selection (open preferences). Note that a closed preference may predict stabilising or directional selection according to the position of the display trait distribution relative to peak preference. (d) Preference functions may vary in strength (grey is weaker), according to the extent of the decrease in attractiveness as displays deviate from peak preference.

We tested two hypotheses about the action of sexual selection through mate choice by relating changes in display traits to changes in mate preferences: (1) The amount of divergence in displays is explained by the strength of mate preferences. This hypothesis predicts that stronger preferences will be associated with greater divergence in display traits. (2) The amount of divergence in display traits is explained by the amount of divergence in mate preferences. This hypothesis makes two predictions: First, greater preference divergence will be associated with greater divergence in display traits. Second, this relationship will be stronger for closed preferences than for open preferences – because the display–preference match should be tighter for closed preferences, which select against deviation from peak preference in both directions, whereas open preferences select against deviation from the peak in only one direction (Rodríguez *et al*. 2006) (see Fig. 2). When possible, we also tested a prediction that relates the two hypotheses above. In some cases, the effect of preference divergence should be greater with stronger preferences (Fig. 1), so that these variables interact in a positive way. However, if the amount of preference divergence determines how much display divergence can occur, there will be a point at which stronger preferences cannot result in any greater Δt (Fig. 1) and the interaction may be negative. Alternatively, with enough time, large preference divergence can result in large display divergence even if preferences are weak (Fig. 1) and the interaction may again be negative. These considerations predict an interaction between the effects of preference strength and preference divergence, which we tested whenever the sample size for each case study allowed constructing a model with the interaction term (see below). Because the interaction can be complex, we focused on testing for its presence, rather than on its sign. We tested these predictions for each case study, and then conducted an overall analysis of effects and effect sizes.

We find a remarkably consistent pattern: Divergence in mating displays is predicted by divergence in mate preferences, whereas the strength of mate preferences has a more subtle effect and is best detected as an interaction with divergence in mate preferences. We argue that failing to capture both of these aspects of sexual selection may lead to underestimation or mischaracterisation of its action, and bias conclusions about its role in important evolutionary processes such as divergent evolution and speciation.

## Methods

**Figure 3:**
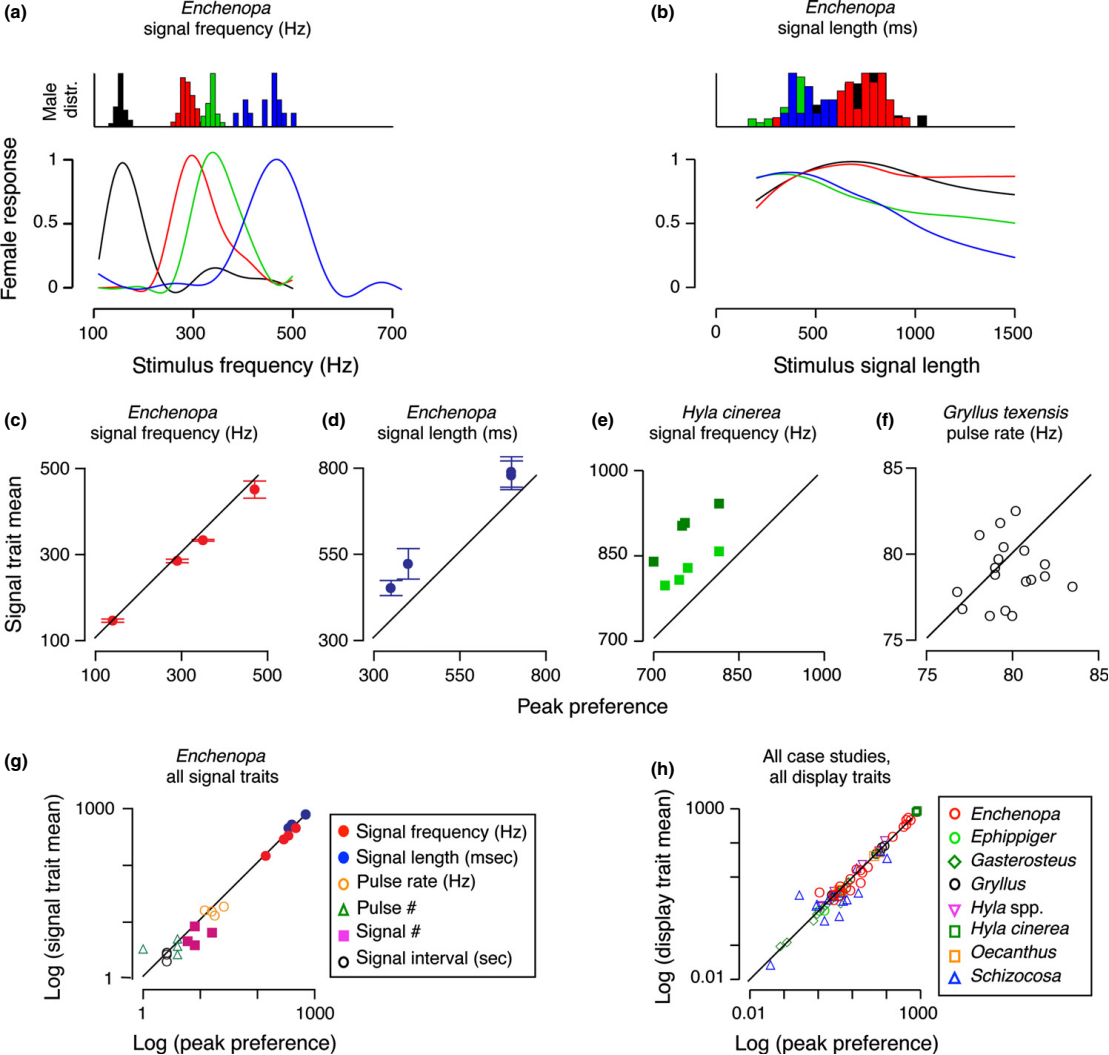
Examples of variation in display–preference correspondence. (a) Close correspondence with strong preferences for *Enchenopa* signal frequency. (b) Lax correspondence with weaker preferences for *Enchenopa* signal length. (c) The pattern from panel a, plotting mean signal and peak preference values. Here and below, the 1 : 1 line indicates perfect correspondence. (d) The pattern from panel b, plotting mean signal and peak preference values. (e) Correspondence among eight *Hyla cinerea* populations, apparent over a pattern of reproductive character displacement; dark green: rough sympatry with closely related *H. gratiosa*; light green: rough allopatry. (f) No correspondence in the panmictic cricket population. (g) Correspondence among species and traits in *Enchenopa*. (h) Correspondence among species and traits across our case studies. In (g) and (h) the axes are dimensionless; shifts along these axes denote changes in trait type, not trait units. (a)–(d) redrawn with permission from Rodríguez *et al*. (2006).

Our tests are based on quantitative descriptions of mate preferences, or mate preference functions. Preference functions relate variation in sexual response to variation in display traits (Wagner *et al*. 1995; Ritchie 1996; Wagner 1998; Gray & Cade 1999; Brooks *et al*. 2005; Rodríguez *et al*. 2006) (Fig. 2). Preference functions can be described as ‘open’ or ‘closed’ according to whether they favour extreme or intermediate display trait values (Fig. 2a,b). Assessing the true shape of a mate preference requires testing sexual response across a biologically relevant range of variation in display trait values. For example, if the range of values tested is too narrow the preference may appear to be open, whereas a broader range might reveal a closed shape. It is therefore advisable to assess preferences along the full natural range of variation in display traits, or even to exceed that range. An excessively broad range, however, might force a closed shape, so the range tested should be biologically relevant. In our case studies, the ranges of display variation used to describe mate preferences either exceeded the natural range for each species to a biologically appropriate extent (e.g. covered the range of the clade; see Fig. 3a,b) or covered the full natural range for the species or population tested (or nearly did so in one case; details in Appendix S1).

When contrasted with the distribution of display trait values in a population, preference functions constitute hypotheses about the form of sexual selection on displays (Fig. 2c). Across species or populations, such comparisons test the influence of past sexual selection on displays (e.g. Ritchie 1996; Rodríguez *et al*. 2006), and comparing preference functions to variation in reproductive success tests the influence of current sexual selection (Sullivan–Beckers & Cocroft 2010). Thus, preference functions are powerful tools for assessing the degree of coevolution between displays and mate preferences, and for testing hypotheses about mate preferences as causes of selection on display traits. As an illustration, in Fig. 3, we show examples of the relationship between mate preferences and male display trait distributions, and of how this relationship can be described with mean display trait and peak preference values (as per Fig. 2). Within and across our case studies, there is an impressive level of display–preference correspondence (Fig. 3). Such close signaller–receiver correspondence, although not universal (e.g. Ryan 1998; Hebets & Maddison 2005), is widespread and suggests a strong potential for sexual selection by mate choice to promote display diversification and thereby contribute to reproductive isolation between diverging populations (West–Eberhard 1983; Boughman 2001; Gerhardt & Huber 2002; Greenfield 2002; Rodríguez *et al*. 2006; Grace & Shaw 2011).

For each case study, we relate changes in preference functions to changes in display traits. Displays included acoustic signals (crickets, frogs, katydids, tree crickets); substrate-borne vibrational signals (treehoppers, wolf spiders); and visual signals (sticklebacks, wolf spiders). Four of the case studies involved multivariate displays encompassing a single modality and corresponding preferences that were described with trials that assessed one or two display traits at a time (*Enchenopa*, *Gryllus* spp., *Hyla* spp., *Oecanthus*); 2 case studies involved multimodal mating displays and preference assessment (*Gasterosteus* and *Schizocosa*); and in 3 case studies a single display trait has a large effect and was the focus of the study (*Ephippiger*, *Hyla cinerea*, panmictic cricket population). Two of the case studies included body size estimates: for *Gasterosteus*, we considered body length as part of the display because females view the entire body of the male during courtship, and there is evidence that it plays a role in mate selection (Nagel & Schluter 1998; Kraak *et al*. 1999; McKinnon *et al*. 2004); for *Schizocosa*, we included cephalothorax width because females could in principle perceive body size and use it in their mating decisions. For simplicity, we refer to all traits as ‘display traits.’

The basic data (see Appendix S2) for each analysis were as follows: (1) the mean value for each display trait for each species or population in each case study, (2) the strength of the corresponding preference (Fig. 2d) and (3) the peak of the corresponding preference (Fig. 2c). Besides preference strength and peak preference, other aspects of the shape of mate preferences may be important, such as the breadth of the peak or the degree of overlap among preferences. However, our goal was to capture the difference in viewpoint that may arise from emphasising the strength of selection vs. divergence in a cause of selection. To this end, preference strength best approximates proxies for the strength of sexual selection that have been used in comparative analysis of the action of sexual selection (see below), and peak preference offers a clear prediction of where mean display traits should be if mate preferences are an important cause of selection on displays. In addition, in our experience preference strength is independent of peak preference (as in our case studies; see below) but correlated with other aspects of preference shape such as breadth (Bailey 2008; Fowler–Finn & Rodríguez 2012a,b; Rodríguez *et al*. 2013a,b). For example, stronger preferences are also narrower and less overlapping (Fig. 3a,b). Although more work is required to assess the generality of such correlations, we consider that the combination of preference strength and peak preference provides a good account of overall variation in preference shape, and one that is ideally suited to our analysis.

### Divergence in displays (Δt) and divergence in peak preferences (Δp)

We obtained dimensionless, unbounded measures of the amount of divergence in display traits (Δt) and peak preferences (Δp) for each species or population in each case study. These measure the distance of a species or population from the group mean in each case study (cf. Arnqvist 1998) and allow us to compare amounts of divergence within and among case studies. Besides mate choice, they are likely influenced by the time since branching from the common ancestor, with older radiations potentially showing more divergence. Δt and Δp varied considerably among case studies (see below), and the species or populations in each study are not sister taxa. However, they belong to the same genus or species complex, and likely represent relatively recent divergence.

We calculated Δt thus: Δt = (trait_mean_ − trait_GrandMean_)/trait_GrandMean_

where trait_mean_ was the mean of each trait in each species or population in the case study; and trait_GrandMean_ was the overall mean for each trait in the case study.

We calculated Δp thus: Δp = (peak − peak_GrandMean_)/peak_GrandMean_

where peak was the peak preference for each trait in each species or population, and peak_GrandMean_ was the overall mean of the peak preferences for each trait in the case study.

Using means in the denominator to calculate Δt and Δp allowed us to generate dimensionless measures sensitive to among-trait differences in the amount of divergence that has occurred, which can then be related to among-trait differences in preference strength and preference divergence. The alternative (to use standard deviations for the denominator) would obscure the among-trait differences in amount of divergence that we wished to capture.

We note that trait_GrandMean_ and peak_GrandMean_ are biased estimates of ancestral states; they would be accurate if taxa were related by a polytomy within each group. This introduces noise into the analysis – some amounts of divergence are overestimated and others are underestimated. This noise makes the hypotheses harder to support (it increases type II error, but not type I error), and in that sense our hypothesis tests are conservative.

We adjusted peak preference estimates to the shape of the preferences. For closed preferences the peak was the trait value eliciting the highest response (Fig. 2a). For open preferences, we defined the peak according to how investment in displays may affect attractiveness. With preferences that plateau (Fig. 2b), investment beyond a certain point does not increase attractiveness. In such cases (*Enchenopa* and *Ephippiger* case studies), we defined the peak at the beginning of the plateau. Even without a plateau (Fig. 2b) there may be diminishing returns if the cost of extreme displays outweighs the increase in attractiveness. In such cases (*Gasterosteus* and *Schizocosa* case studies) we defined the peak as the display trait value at which female response was 75% of the maximum (see dotted horizontal line in Fig. 2b). To assess if this criterion could bias our analysis, we evaluated the effect of using other response levels: higher levels simply made the Δt∼Δp relationship shallower (Fig. S1 in Appendix S3), but did not affect the outcome of the analyses (Table S1 in Appendix S3).

### Preference strength

We obtained a dimensionless, unbounded measure of preference strength. The strength of sexual selection is determined by variance in reproductive success and depends largely on mating system (Shuster & Wade 2003). Because mating systems are consistent within case studies (see Appendix S1), we expect the strength of sexual selection to be related to preference strength for each case study. Our approach thus controls for potentially confounding variation in mating systems. We estimated preference strength with the square of the Coefficient of Variation (CV^2^) of female response scores across the range of trait values for each species/population (Schluter 1988; Fowler–Finn & Rodríguez 2012a,b; Rodríguez *et al*. 2013a,b). For the *Gasterosteus* and *Schizocosa* case studies (open preferences), we estimated the CV^2^ from the Sum of Squares of a linear regression of female response on the male trait, thus: CV^2^ = (√SS/trait_mean_)^2^.

Preference strength and Δp were unrelated to each other: the correlation between the absolute value of Δp and preference strength varied across case studies (Table 1), with an overall median of *r* = −0.03.

**Table 1:** Relationship between the amount of divergence in peak preferences (Δp) and preference strength, and the amount of divergence in display traits (Δt). We highlight the Δp term for models with signed Δ values, and the preference strength term for models with absolute Δ values (see Statistical analysis). Significant or marginally significant terms in bold

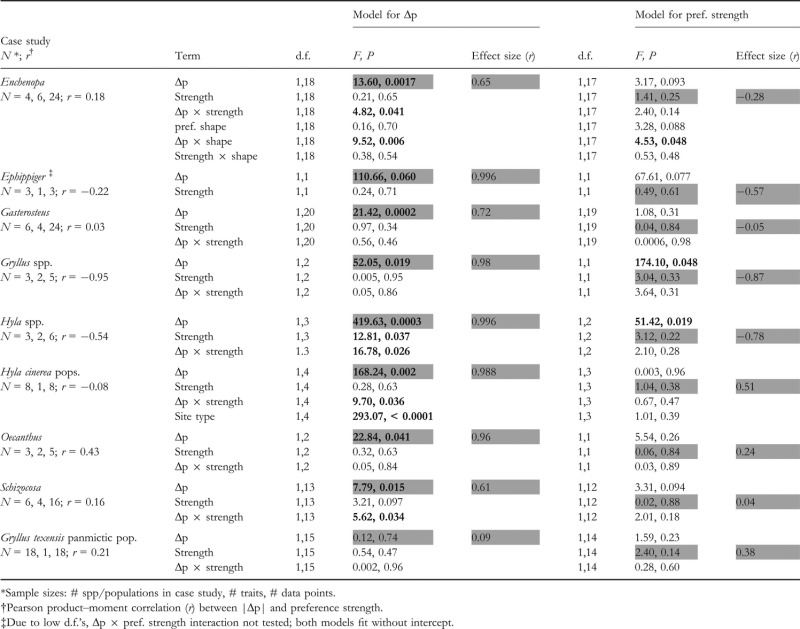

### Statistical analysis

We conducted all analyses in JMP 7.0.1 (SAS Institute, NC, USA). In each case study, the dependent variable was Δt, and the independent variables were Δp, preference strength, and their interaction when the sample size allowed including it in the statistical model (Table 1). Note that the prediction is for the Δp × preference strength interaction to be present, rather than for it to be of a particular sign (i.e. positive or negative; see above). The *Enchenopa* case study had open and closed preferences, so we also tested the effect of preference shape and its interaction with Δp and preference strength (Table 1). In the other case studies, preferences were either all closed or all open.

**Figure 4:**
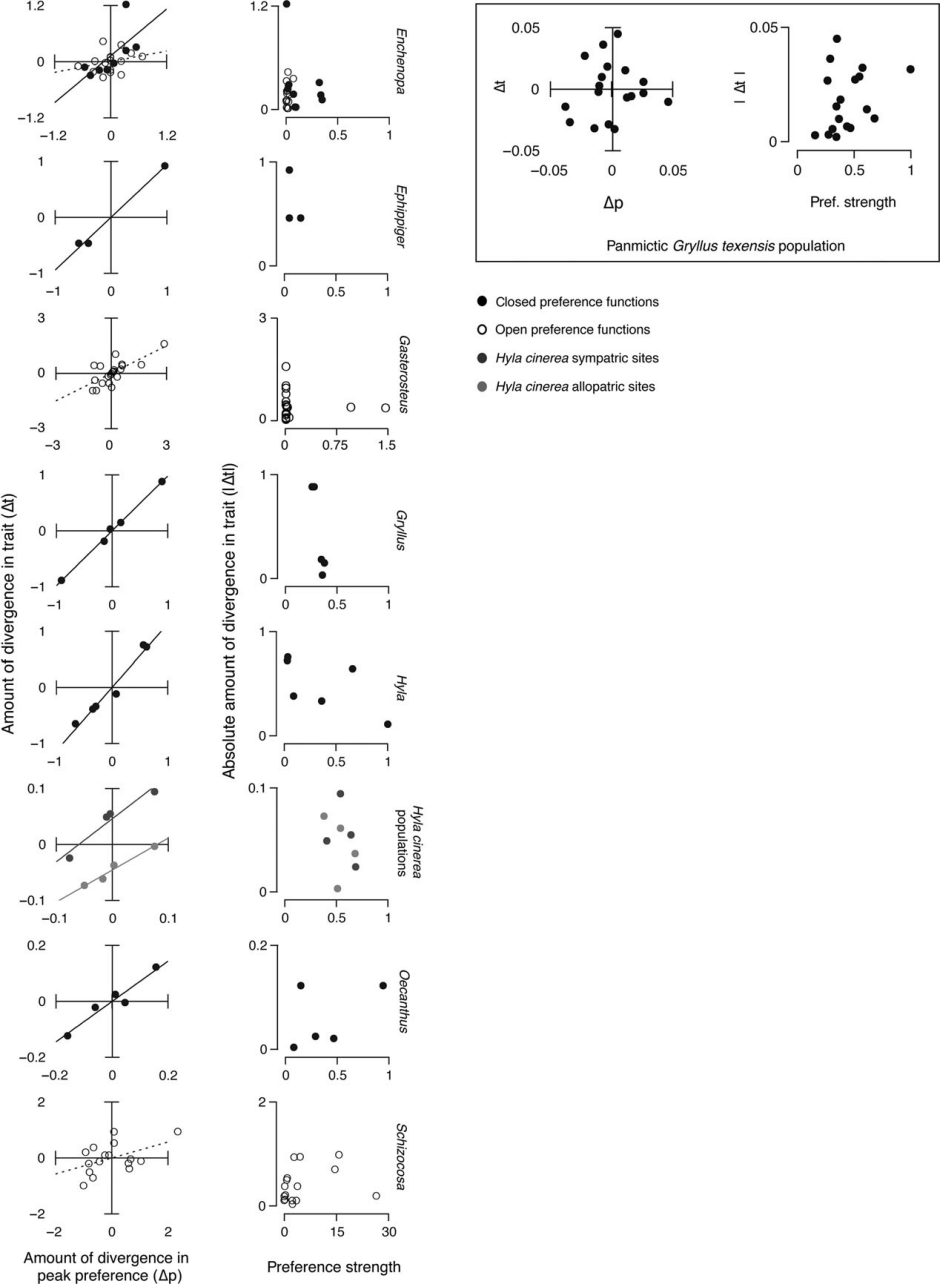
Relationship between the amount of divergence in display traits (Δt) and the amount of divergence in peak preferences (Δp, left column), or preference strength (right column) for our case studies. The relationship between Δt and Δp was consistently positive and strong, except for the panmictic cricket population (inset). By contrast, there was no relationship between Δt and preference strength. Note the much lower magnitude of Δt and Δp values for the panmictic cricket population (inset).

We ran the models in two different ways to optimise the tests for Δp and for preference strength (Table 1). This is because Δt and Δp are signed, which is appropriate for relating changes in preferences to changes in displays (Figs 1 and 4), but inappropriate for relating preference strength to changes in displays because stronger preferences are predicted to result in greater divergence in any direction. Thus, the model for Δp used the signed values, whereas the model for preference strength used absolute Δt and Δp values. We retained the term for preference strength in the model for Δp to test for the Δp × preference strength interaction, which relates the effects of Δp and preference strength to each other (see above). Removing those two terms from the models testing for the effect of Δp did not alter the outcome of the analyses (see below). We fit the model for Δp without the intercept because the Δt∼Δp relationship is constrained to pass through the origin (Fig. 4). We fit the model for preference strength with the intercept – either formulation yielded qualitatively the same results for most case studies, but the intercept model dealt better with the data in the case studies featuring geographic variation (*H. cinerea* and panmictic cricket population). This double testing for each case study increases the risk of spurious significance (Rice 1989), but corrections against it compromise statistical power (Moran 2003; Nakagawa 2004). We dealt with this problem by assessing table-wide patterns of significance (Moran 2003). We also estimated effect sizes for Δp and preference strength as correlation coefficients (*r*) from the *F* ratio of their term in the model (Table 1), thus: *r* = √[*F*/(*F* + d.f._error_)] (Rosenthal 1991; Nakagawa & Cuthill 2007).

In the above analyses (Table 1), for each case study we use data from different species and traits (e.g. signal frequency and length) as independent data points although they are likely correlated (e.g. in *Enchenopa*, longer signals are lower in frequency; Cocroft *et al*. 2010). Our rationale for this approach was as follows: we expect the relationships between preferences and display traits to be independent among traits; for instance, the preference strengths and peaks pertaining to signal frequency are likely to be independent of the strengths and peaks pertaining to signal length (e.g. Fig. 3a–d). To test this expectation, we used linear mixed models including species or population and trait as random effects, using the REML method in JMP. Three of the case studies involved a single display trait (*Ephippiger*, *H. cinerea* and panmictic cricket population) and so we only entered the term for species or population as a random effect. If our expectation is correct, the terms for species or population and for trait should have no effect in these models. The REML method in JMP provides variance component estimates rather than significance tests, and so we checked whether the confidence intervals for the variance component overlapped zero. In all cases, the confidence intervals for the terms for species or population and for trait overlapped zero (or the component was nearly exactly zero in the case of the *H. cinerea* population term). We thus consider that our expectation of independence in the relationships among preferences and displays traits is justified, and we used models without the above random effects (Table 1).

Another potential concern is that Δt and Δp values may scale with the trait means, given that we standardised with the grand mean for each trait in each case study (see above). If Δt and Δp are positively correlated with the mean, then traits with larger means might have a larger influence on the analyses than traits with smaller means. In three of our case studies, there was no risk of this, as they involved a single display trait (*Ephippiger*, *H. cinerea* and panmictic cricket population). For the other case studies, we tested for this possibility by assessing the relationship between trait means (as the independent variable) and our estimates for Δt, Δp and preference strength as dependent variables in separate analyses. In the 6 case studies involving more than one display trait, there was never a significant relationship between trait means and our estimates for Δt (*P* ≥ 0.31) or for Δp (*P* ≥ 0.33); in four of the case studies the relationship between trait mean and preference strength was also non-significant (*P* ≥ 0.35), but in 2 case studies it was significant or marginally significant (*Gryllus* spp.; *P* = 0.03; *Enchenopa*; *P* = 0.07, although the latter relationship was negative). Overall, the criterion of table-wide significance (Moran 2003; see above) suggests that those two (of 18) tests that were significant may be spurious, and that we do not have a problem of scaling with trait means.

## Results

We found a pronounced difference in how the amount of display divergence (Δt) relates to preference strength and to the amount of preference divergence (Δp). The relationship between preference strength and Δt was never significant (Table 1; Fig. 4). By contrast, the relationship between Δp and Δt was significant or marginally significant in eight of the 9 case studies – i.e. in all but the panmictic cricket population (Table 1; Fig. 4). The models that were optimised to test for the effect of Δp (Table 1) also included terms for preference strength and its interaction with Δp (see below). Excluding these two terms from these models yielded the same results: The term for Δp remained significant in 7 case studies (*P* ≤ 0.0075) and was marginally significant (*P* = 0.059) only for the *Schizocosa* case study; as above, the term for Δp remained non-significant for the panmictic cricket population (*P* = 0.72).

**Figure 5:**
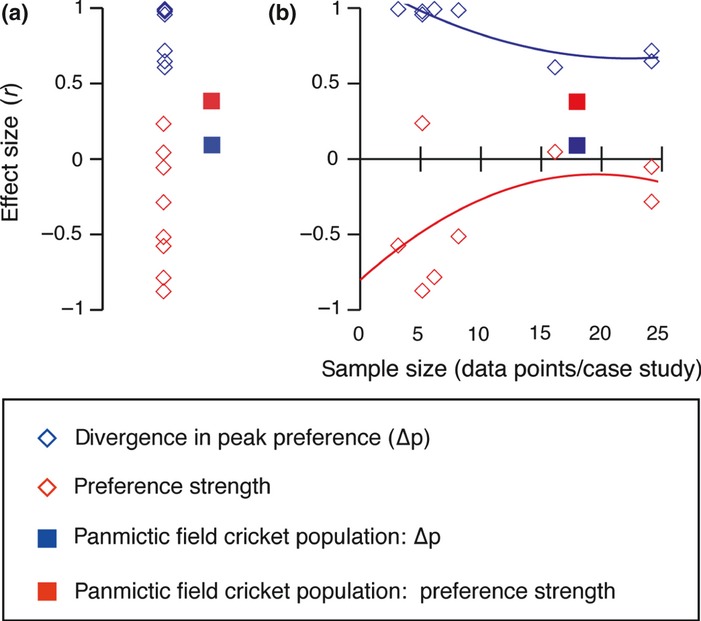
Effect sizes (*r*) for the relationship between the amount of divergence in peak preferences (Δp) or preference strength and the amount of divergence in display traits (Δt). Data points show effect size estimates for each trait in each case study. (a) Effect sizes for Δp were greater than for preference strength. (b) Effect sizes varied with the sample size of each case study (*N* = # data points in case study = # traits × # taxa in case study), but remained consistently large and positive for Δp, and either small or large negative for preference strength. Fitted lines are quadratic functions that asymptote at a larger effect size for Δp than for preference strength.

We further compared how Δt relates to Δp and preference strength in terms of the effect size of the relationships. We found that the effect sizes for the Δt∼Δp relationship were significantly greater than for the Δt∼preference strength relationship (Welch anova allowing for unequal variances: *F*_1,8.9314_ = 53.97, *P* < 0.0001; Fig. 5a). This pattern remained when we used the absolute value of the effect sizes (Welch anova: *F*_1,9.892_ = 9.89, *P* = 0.022). We also found that these effect sizes were influenced by the sample size of each case study, with smaller *N* case studies likely overestimating effect sizes (Fig. 5b). Across 8 case studies (conservatively excluding the panmictic cricket population), the correlation between *N* and the effect size for Δp was *r* = −0.89, *P* = 0.0031; for preference strength, it was *r* = −0.66, *P* = 0.073. Nevertheless, effect sizes remained consistently stronger for Δp than for preference strength (Fig. 5b). In short, we found that the effect sizes for the Δt∼Δp relationship were always strong and positive, whereas the effect sizes for the Δt∼preference strength relationship were either weakly positive or negative (Figs 4 and 5).

**Figure 6:**
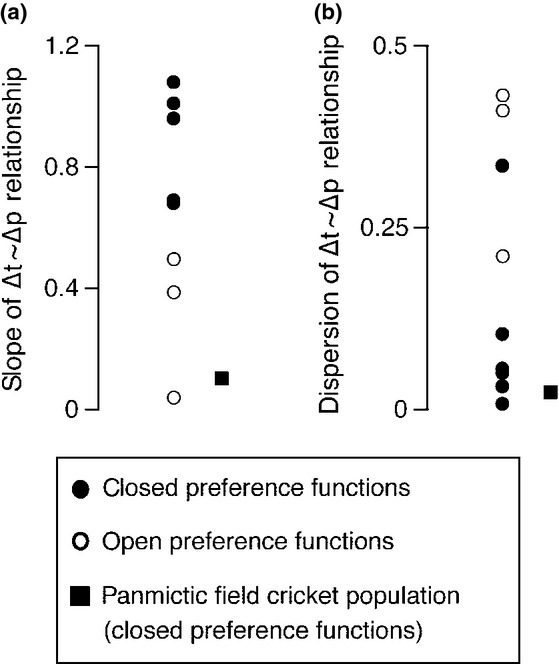
The relationship between the amount of divergence in peak preferences (Δp) and display traits (Δt) was steeper and less disperse for closed preferences than for open preferences. (a) Difference in slope: *F*_1,7_ = 19.12, *P* = 0.0033. (b) Difference in dispersion around trend line, measured with the Standard Error of the Estimate (SEE = √MS_error_): *F*_1,7_ = 8.81, *P* = 0.021.

An additional feature of the Δt∼Δp relationship was that it was both steeper and less disperse for closed preferences than for open preferences (Fig. 6).

In seven of the case studies, sample sizes allowed us to test for a statistical interaction between the effects of Δp and preference strength on Δt. In four of these 7 case studies, this interaction was significant (Table 1). The interaction was positive in 2 case studies (*H. cinerea* populations and *Schizocosa*; estimate = 1.9 and 0.1 respectively), and negative in the other two (*Enchenopa* and *Hyla* spp.; estimate = −3.45 and −0.64 respectively).

In the panmictic cricket population, any site differences reflect sampling variation. Therefore, Δt and Δp should be small, and there should be no relationship between them or between preference strength and Δt. We found that Δp and Δt showed an order of magnitude less divergence than the least divergent of the other case studies (Fig. 4). There was no significant relationship between either Δp or preference strength with Δt (Table 1; Fig. 4). Effect sizes were negligible for Δp and medium positive for preference strength (Fig. 5).

## Discussion

We evaluated the potential of two parameters of sexual selection by mate choice to explain divergence in mating displays. Stronger mate preferences were not associated with greater display divergence in a simple way. By contrast, more divergent mate preferences were closely associated with greater display divergence, especially for preferences of closed shape. This pattern supports the notion that preference strength and preference divergence play different roles in diversification. Preference divergence determines the amount of divergence in displays, whereas preference strength determines the rate of evolution and the closeness of the display–preference match. Thus, a preference with a peak near the ancestral state can only produce little divergence, no matter how strong it is, whereas a more divergent preference can cause greater diversification even if weak (Fig. 1). Consequently, the best chance of detecting an effect of preference strength in our analysis is via the interaction with preference divergence; this interaction was significant in four of the 7 case studies in which it was testable.

Our findings suggest that failing to capture the different aspects of the action of sexual selection may lead to underestimation of its role in processes such as adaptation and divergence. For example, if we were to rely solely on preference strength, we would conclude that sexual selection by mate choice has very little to do with display trait diversification in our case studies, whereas incorporating preference divergence in our analysis reveals quite the opposite. This concern has implications for the study of the role of sexual selection in speciation, a topic that remains controversial due to mixed results in spite of decades of theoretical and empirical work. There is, on one hand, a widespread trend for sexually selected traits to be the most divergent aspects of the phenotypes of closely related species (West–Eberhard 1983; Eberhard 1985; Andersson 1994; Coyne & Orr 2004; Mendelson & Shaw 2005; Arnegard *et al*. 2010; Safran *et al*. 2012). And there are also robust examples of sexual selection making stronger contributions to the evolution of reproductive isolation than natural selection (Gray & Cade 2000; Wilson *et al*. 2000; Masta & Maddison 2002; Svensson *et al*. 2006; Boul *et al*. 2007; Funk *et al*. 2009; Sota & Tanabe 2010). But, on the other hand, there is only mixed support for the prediction that clades wherein sexual selection has a stronger influence should exhibit higher speciation rates (Coyne & Orr 2004; Panhuis *et al*. 2001; Ritchie 2007; Ritchie *et al*. 2007; Seddon *et al*. 2008; Kraaijeveld *et al*. 2010). We suggest that this ambiguity may arise in part because tests of the role of sexual selection in speciation have not accounted both for the strength of selection as well as for how divergent selection is. Comparative analyses may have thus underestimated the diversifying effect of sexual selection, perhaps drastically. Divergence in mating displays and preferences does not equal speciation; there can be, for instance, considerable within-species divergence in polymorphic or phenotypically plastic sexual traits (West–Eberhard 2003). Nonetheless, when speciation occurs, the underlying causes of reproductive isolation often involve traits such as displays and preferences (e.g. Gray & Cade 2000; Wilson *et al*. 2000; Boughman 2001; Masta & Maddison 2002; Boughman *et al*. 2005; Svensson *et al*. 2006; Boul *et al*. 2007; Funk *et al*. 2009; Stelkens & Seehausen 2009; Sota & Tanabe 2010). We therefore suggest that using quantitative descriptions of the causes of sexual selection, such as mate preference functions, and incorporating measures of the amount of divergence in the form of sexual selection as well as of the strength of sexual selection, will improve the power of predictions about the rate of speciation in comparative studies of the role of sexual selection in speciation. It may also improve tests of the relationship between divergence in mate preferences and mating displays and reproductive isolation.

The remarkable consistency of our results across case studies suggests that we have identified a robust pattern about the action of sexual selection. However, the number of studies that can be analysed with our approach is small, and should be increased in the future. One interesting avenue to expand the empirical framework we develop here will be to implement it with multivariate and multimodal approaches (e.g. Brooks *et al*. 2005; Hebets & Papaj 2005), as well as to refine it by incorporating consideration of the amount of genetic variation available for the different aspects of selection to act upon (Chenoweth *et al*. 2010). It will also be important to include traits that function at stages of the reproductive process beyond pair formation (Eberhard 2011). Another fruitful broadening of the framework presented here will be to consider different ways in which selection may be divergent. Our analyses encompass two such ways: We have focused on a single cause of selection (mate choice) that may be divergent by favouring different display trait values (among-species variation in Δp), and by doing so to different extents (among-trait variation in preference strength). The latter may also take more extreme forms, so that a single cause of selection (e.g. mate choice) may target qualitatively different traits in different species (e.g. signal rate vs. length in different, closely related species; Schul & Bush 2002). Similarly, male–male competition may also target qualitatively different traits (e.g. body size vs. coloration; Lackey & Boughman 2013). Indeed, such qualitative shifts in the targets of selection can be important in the evolution and divergence of complex displays (West–Eberhard 1983). Yet another way in which selection can be divergent involves differences in which cause of selection targets different traits. For instance, some traits may diverge mainly because of mate choice and others because of direct male–male competition.

In conclusion, we suggest a view of the action of selection that focuses not only on the strength of selection but also on quantitative descriptions of how divergent selection is, as we do here with mate preferences. Perhaps the greatest challenge in this endeavour will lay in putting characterisations of natural and sexual selection on the same footing – i.e. generating ‘ecological performance functions’ comparable to mate preference functions, to then relate each to observed patterns of trait diversification and species divergence. This expanded view may revolutionise our understanding of the action of ecological and sexual mechanisms of selection.
