# Supplementary material for: Diversification under sexual selection: the relative roles of mate preference strength and the degree of divergence in mate preferences
Source: Ecol Lett. 2013 Jul 1;16(8):964–74. doi: 10.1111/ele.12142 (PMC3757319; doi:10.1111/ele.12142)
Supplement: Supplementary file 1 [file ele0016-0964-SD1.doc]

**Appendix S1 *Case studies***

***Enchenopa binotata* species complex (Hemiptera: Membracidae).** Speciation in this clade of phloem–feeding insects involves colonization of novel environments and divergence in communication systems (Cocroft *et al*. 2008). Pair formation involves duets of plant–borne vibrational signals: males produce advertisement signals and receptive females respond with their own signals. Sources of selection on signals include mate preferences and plant signal–transmission properties (Rodríguez *et al*. 2004, 2006; McNett & Cocroft 2008). Of these, mate preferences make the stronger contribution (Sullivan–Beckers & Cocroft 2010). Data (see Appendix S2) were obtained from a study with four syntopic members of the complex from Missouri, USA (Rodríguez *et al*. 2006). Experiments used laser vibrometry and vibrational playback of stimuli covering the range in the species complex, thus exceeding the natural range of any one species (e.g., see Fig. 3a and b; Rodríguez *et al*. 2006; Cocroft *et al*. 2010). Preference functions were described with random regression and non–parametric cubic splines (Rodríguez *et al*. 2006).

***Ephippiger* bushcrickets (Orthoptera, Tettigoniidae).** *Ephippiger* males produce a pulsed acoustic signal to attract females. Some populations of *E. ephippiger* produce monosyllabic signals with a single pulse, whereas others produce polysyllabic signals. *Ephippiger terrestris* males produce monosyllabic signals. Data on male signals and female preferences (see Appendix S2) were obtained from published work wherein preference functions were determined using virgin females in the laboratory; female choice trial data were obtained by repeatedly testing individual females using alternate stimuli sets that covered the range from monosyllabic to polysyllabic signals, thus exceeding the natural range of any one population (Ritchie 1996, 2000). Preference functions were closed.

***Gasterosteus aculeatus* threespine stickleback complex.** This complex shows rapid divergence throughout its holartic range, and post–pleistocene speciation has occurred repeatedly (Schluter & McPhail 1992; McPhail 1994; Rundle *et al.* 2000; McKinnon & Rundle 2002). Male secondary sexual traits and female preferences show extensive diversification, and the repeated nature of such evolution makes this a model system for studying diversification. Signaling occurs in multiple modalities including olfactory and visual (color, size, shape, display traits) (Boughman 2006; Rafferty & Boughman 2006). Although males provide all parental care, females have strong mate preferences. The visual environment exerts selection on male color and female color preference (Boughman 2001), and color is condition–dependent in the limnetic species, consistent with the presence of strong color preferences (Boughman 2007). Reproductive isolation is strong and consists primarily of sexual isolation (Boughman 2001) coupled with ecologically–dependent postmating isolation (Schluter 1995; Hatfield & Schluter 1999; Gow *et al.* 2007). Data (see Appendix S2) were obtained from no–choice mating trials conducted within and between multiple populations of the limnetic, benthic, and marine species; thus, the range of displays evaluated exceeded the natural range of any one population (Boughman *et al.* 2005; Boughman 2007; Head *et al.* 2009). We describe mate preferences with a continuous measure of response to male courtship (examining the male’s nest) (Kozak *et al.* 2009). We explored preference for the area and intensity of red color, male courtship display, and male length (see Appendix S2).

***Gryllus* field crickets (Orthoptera: Gryllidae).** Male field crickets produce a long distance calling song to attract females for mating. We obtained comparable published and unpublished trait and preference data (see Appendix S2) for pulse rates for *Gryllus texensis*, *G. rubens*, and *G. integer* (Hedrick & Weber 1998; Gray & Cade 2000; Izzo & Gray 2004; D.A. Gray, unpubl.). Data on numbers of pulses per unit of song (chirp/trill) along with preference data were obtained for *Gryllus texensis* and *G. integer* [note that *G. texensis* was formerly studied under the name *G. integer*] (Hedrick & Weber 1998; Gray & Cade 1999); all studies were laboratory based. In each of these species, both pulse rates and numbers of pulses per chirp/trill are under stabilizing selection with closed preference functions. The range of stimuli tested either exceeded the natural range (*G. integer*) or covered the full natural range (*G. texensis* and *G. rubens*). Note that the study on *G. integer* refers to “syllable number” (Hedrick & Weber 1998; see Appendix S2) whereas the study on G. texensis refers to “pulses per trill” (Gray & Cade 1999; see Appendix S2). Although these are homologous traits, we have retained the terminology used by each paper, in order to facilitate going back to the original papers to look for the source data (see Appendix S2). Mitochondrial DNA data show that *G. texensis* and *G. rubens* are sister taxa; *G. integer* is more distant within the larger North American *Gryllus* clade (Gray *et al.* 2006, 2008; D.A. Gray and D.B. Weissman, unpubl.).

***Gryllus texensis* field crickets (Orthoptera: Gryllidae) panmictic population.** This species ranges from central Texas across the southern gulf states to western Florida, USA. Males produce a trilled calling song, typically about 80 pulses/sec at 25 C and about 45 pulses/trill (Gray & Cade 1999; Izzo & Gray 2004). Prior work has shown that crickets from 19 localities do not show systematic changes in either song or preference (see Appendix S2) with respect to general geography or sympatry/allopatry with the sister species, *G. rubens*; further, mitochondrial DNA sequence data from 23 localities showed no differentiation or isolation by distance, suggesting one large pan–mictic population (Gray *et al.* 2008). The range of stimuli used to describe preference functions covered the full natural range.

***Hyla* treefrogs (Anura: Hylidae).** Male *Hyla* tree frogs use acoustic signals to attract females. Data (see Appendix S2) were obtained from published work (Wollerman 1998; Bush *et al.* 2002; Gerhardt 2004). No–choice and two–choice methods for describing preference functions were combined in this analysis. While all tests were conducted at the same temperature, methodological details varied slightly among studies. Preference or response functions were obtained for two signal traits (pulse rate and dominant frequency) for three species (*H. versicolor*, *H. chrysoscelis*, and *Dendropsophus ebraccatus* [formerly *Hyla ebraccata*]). The range of stimuli tested either exceeded the natural range (*H. chrysoscelis* in Bush *et al*. 2002) or covered the full natural range (*H. chrysoscelis* and *H. versicolor* in Gerhardt 2005; *D. ebraccatus* — for the latter the range covered percentiles 5 to 95 of the natural range; Wollerman 1998). *Hyla versicolor* is the tetraploid sister species of *H. chrysoscelis*, while *D. ebraccatus* is more distantly related.

***Hyla cinerea* treefrogs (Anura: Hylidae).** This species ranges from southern Texas to Delaware, USA. In the eastern part of its range it overlaps with its sister species, *H. gratiosa*, and allopatric and sympatric populations show reproductive character displacement (Höbel & Gerhardt 2003). Males produce acoustic signals to attract females. Females show strongest preferences for call frequency. Preference functions were described with non-parametric cubic splines using data from (Höbel & Gerhardt 2003). Data are from 8 populations from Texas, Louisiana, Mississippi, Alabama, Georgia and South Carolina (see Appendix S2). Females were collected in amplexus to assure receptivity to sexual signals. Trials were conducted in the field, using a portable playback arena. Preferences were described using two–choice playback trials that tested a 900 Hz standard signal against lower (600, 700, 800Hz) and higher (1000, 1100 1200Hz) alternatives; 10-23 females were tested in each population. The range of stimuli tested either exceeded the natural range of any one population. A minimum of 30 males were recorded in each populations to obtain data on signal trait variation.

***Oecanthus* tree crickets (Orthoptera: Gryllidae).** *Oecanthus* are widespread in North America, living on shrubs and trees in a variety of habitats. Males signal to advertise their presence and receptive females walk toward preferred signals (Walker 1957; Brown *et al.* 1996). Preference functions were generated for females of three different species: *O. forbesi*, *O. nigricornis,* and *O. quadripunctatus.* Females were field–caught as nymphs and adults during the summer and fall of 2011 and held in captivity until tested. Preferences were characterized for signal pulse rate and dominant frequency. Preferences were examined by creating 5 evenly–spaced synthetic stimuli spanning a window of 10 pulses/sec (pulse rate series) or ca. 900 Hz (frequency series) centered around the population mean. Non–focal signal traits were held constant. In each trial, females were presented with pairs of stimuli that were one step apart in the series. All trials were conducted at 25±1 C in an arena consisting of a 1.2 m–radius acoustic foam ring with two Genelec 1060 speakers placed 1 m apart. The speakers were angled inward at 110° so that both faced a central point 67 cm in front of them. This minimized potential cancellation between speakers when the female was at the central point. Females were placed on the central point under a plastic cup and allowed to acclimate for 30 sec. Then, both stimuli were played simultaneously, each from a randomly assigned speaker. If a female made contact with the speaker within 120 sec, it was scored as a response to the stimulus. Preference was scored as the percentage of total trials in which females responded to a given stimulus (see Appendix S2). The range of stimuli tested either exceeded the natural range of any one species.

***Schizocosa* wolf spiders (Araneae: Lycosidae).** The genus *Schizocosa* is a young lineage, with species divergence hypothesized to have occurred since the last glaciers (Stratton 2005). Among the 23 described North American species, tremendous variation exists in male secondary sexual traits and associated behavior – providing a model system for exploring the evolution of rapid and extreme diversification. Sexually dimorphic ornamentation consists of pigmentation and/or brushes of setae on segments of the forelegs (reviews: Stratton 2005; Framenau & Hebets 2007; Vaccaro *et al.* 2010). In addition to ornamentation, the forelegs are frequently waved or tapped vigorously during courtship. Numerous studies have demonstrated condition-dependence of *Schizocosa* foreleg ornamentation — e.g., *S. ocreata* (Uetz *et al.* 2002); *S. uetzi* (Shamble *et al.* 2009); *S. floridana* (Rundus *et al.* 2011); *S. bilineata* (Berns 2011) — as well as the importance of courtship rate for mating success — e.g., *S. floridana* (Rundus *et al.* 2011); *S. retrorsa* (Rundus *et al.* 2010); *S. uetzi* (Shamble *et al.* 2009); *S. stridulans* (Hebets *et al.* 2011); *S. bilineata* and *S. crassipalpata* (Berns 2011). We used data from six *Schizocosa* species (see Appendix S2) generated from laboratory mating trials. Individuals used in the trials were virgins collected as subadults (see Appendix S2 for collection locales). Data for these analyses represent subsets of data intended for alternative studies and thus diet treatments vary across species (see Appendix S2). Male traits examined included (i) tibial pigmentation (mean darkness value; for all but the non–sexually dimorphic *S. crassipalpata*), (ii) brush area (mm^2^, for *S. bilineata* and *S. crassipes*), (iii) courtship rate (# visual displays/time; for all but *S. crassipes*), and (iv) size (cephalothorax width - CW) (see Appendix S2). Of these traits, females use CW the least in making mating decisions, and preferences were weak; we included this trait in the analysis for completeness: lack of a relationship between this trait and female behavior makes the analysis more conservative for the hypotheses that we test. The range of displays evaluated covered the full range for each species. To obtain preference peaks, we converted categorical responses (yes/no) to 0 or 1 values and used linear regressions.

**REFERENCES**

Berns, M.D. (2011). *Exploring sources of selection on the multimodal courtship displays of two sister species of wolf spiders:* Schizocosa crassipalpata *and* Schizocosa bilineata. Master’s Thesis. University of Nebraska, Lincoln.

Boughman, J.W. (2001). Divergent sexual selection enhances reproductive isolation in sticklebacks. *Nature*, 411, 944–948.

Boughman, J.W. (2006). Speciation in sticklebacks. In: (*The Biology of the Three-spined Stickleback*) {eds. Östlund–Nilsson, S., Mayer, I. & Huntingford, F.A.} CRC Press, Boca Raton, FL, USA, pp 83–126.

Boughman. J.W. (2007). Condition–dependent expression of red colour differs between stickleback species. *J. Evol. Biol.*, 20, 1577–1590.

Boughman, J.W., Rundle, H.D. & Schluter, D. (2005). Parallel evolution of sexual isolation in sticklebacks. *Evolution*, 59, 361–373.

Brown, W.D., Wideman, J., Andrade, M.C.B., Mason, A.C. & Gwynne, D.T. (1996). Female choice for an indicator of male size in the song of the black-horned tree cricket, O*ecanthus nigricornis* (Orthoptera: Gryllidae: Oecanthinae). *Evolution*, 50, 2400–2411.

Bush, S.L., Gerhardt, H.C. & Schul, J. (2002). Pattern recognition and call preferences in treefrogs (Anura: Hylidae): a quantitative analysis using a no-choice paradigm. *Anim. Behav.*, 63, 7–14.

Cocroft, R.B., Rodríguez, R.L. & Hunt, R.E. (2010). Host shifts and signal divergence: mating signals covary with host use in a complex of specialized plant–feeding insects. *Biol. J. Linn. Soc.*, 99, 60–72.

Cocroft, R.B., Rodríguez, R.L. & Hunt, R.E. (2008). Host shifts, the evolution of communication, and speciation in the *Enchenopa binotata* species complex of treehoppers. In: (*Specialization, speciation, and radiation: the evolutionary biology of herbivorous insects*) {ed. Tilmon, K.} University of California Press, Berkeley, CA, USA, pp 88–100.

Framenau, V.W. & Hebets, E.A. (2007). A Review of Leg Ornamentation in Male Wolf Spiders, with the Description of a New Species from Australia, *Artoria schizocoides* (Araneae, Lycosidae). *J. Arachnol.*, 35, 89-101.

Gerhardt, H.C. (2004) Acoustic spectral preferences in two cryptic species of grey treefrogs: implications for mate choice and sensory mechanisms. *Anim. Behav.*, 70, 39–48.

Gow, J.L., Peichel, C.L. & Taylor, E.B. (2007). Ecological selection against hybrids in natural populations of sympatric threespine sticklebacks. *J. Evol. Biol.*, 20, 2173–2180.

Gray, D.A., Barnfield, P., Seifried, M. & Richards, M.H. (2006). Molecular divergence between *Gryllus rubens* and *Gryllus texensis*, sister species of field crickets (Orthoptera: Gryllidae). *Can. Ent.*, 138, 305–313.

Gray, D.A. & Cade, W.H. (1999). Quantitative genetics of sexual selection in the field cricket, *Gryllus integer.* *Evolution*, 53, 848–854.

Gray, D.A. & Cade, W.H. (2000). Sexual selection and speciation in field crickets. *Proc. Natl. Acad. Sci. USA*, 97, 14449–14454.

Gray, D.A., Huang, H. & Knowles, L.L. (2008). Molecular evidence of a peripatric origin for two sympatric species of field cricket (*Gryllus rubens* and *G. texensis*) revealed from coalescent simulations and population genetic tests. *Mol. Ecol.*, 17, 3836–3855.

Hatfield, T. & Schluter, D. (1999). Ecological speciation in sticklebacks: environment–dependent hybrid fitness. *Evolution*, 53, 866–873.

Head, M.L., Price, E.A. & Boughman, J.W. (2009). Body size differences do not arise from divergent mate preferences in a species pair of threespine stickleback. *Biol. Lett.*, 5, 517–520.

Hebets, E.A., Stafstrom, J.A., Rodríguez, R.L. & Wilgers, D.J. (2011). Enigmatic ornamentation eases male reliance on courtship performance for mating success. *Anim. Behav.*, 81, 963–972.

Hedrick, A. & Weber, T. (1998). Variance in female responses to the fine structure of male song in the field cricket, *Gryllus integer*. *Behav. Ecol.*, 9, 582–591.

Höbel, G. & Gerhardt, H.C. (2003). Reproductive character displacement in the acoustic communication system of green tree frogs (*Hyla cinerea*). *Evolution*, 57, 894–904.

Izzo, A.S. & Gray, D.A. (2004). Cricket song in sympatry: examining reproductive character displacement and species specificity of song in *Gryllus rubens*. *Ann. Ent. Soc. Am.*, 97, 831–837.

Kozak, G.M., Reisland, M. & Boughman, J.W. (2009). Sex differences in mate recognition for species with mutual mate choice. *Evolution*, 63, 353–365.

McKinnon, J.S. & Rundle, H.D. (2002). Speciation in nature: the threespine stickleback model systems. *Trends Ecol. Evol.*, 17, 480–488.

McNett, G.D. & Cocroft, R.B. (2008). Host shifts favor vibrational signal divergence in *Enchenopa binotata* treehoppers. *Behav. Ecol.*, 19, 650–656.

McPhail, J.D. (1994). Speciation and the evolution of reproductive isolation in the sticklebacks (*Gasterosteus*) of south–western British Columbia. In: (*The evolutionary biology of the threespine sticklebacks*) {eds. Bell, M.A. & Foster, S.A.} Oxford University Press, Oxford, UK, pp 399–437.

Rafferty, N. & Boughman, J.W. (2006). Olfactory mate recognition in a sympatric species pair of threespine sticklebacks. *Behav. Ecol.*, 17, 965–970.

Ritchie, M.G. (1996). The shape of female mating preferences. *Proc. Natl. Acad. Sci. USA*, 93, 14628–14631.

Ritchie, M.G. (2000). The inheritance of female preference functions in a mate recognition system. *Proc. R. Soc. Lond. B*, 267, 327–332.

Rodríguez, R.L., Ramaswamy, K. & Cocroft, R.B. (2006). Evidence that female preferences have shaped male signal evolution in a clade of specialized plant–feeding insects. *Proc. R. Soc. B*, 273, 2585–2593.

Rodríguez, R.L., Sullivan, L.E. & Cocroft, R.B. (2004). Vibrational communication and reproductive isolation in the *Enchenopa binotata* species complex of treehoppers (Hemiptera: Membracidae). *Evolution*, 58, 571–578.

Rundle, H.D., Nagel, L., Boughman, J.W. & Schluter, D. (2000). Natural selection and parallel speciation in sympatric sticklebacks. *Science*, 287, 306–308.

Rundus, A.S., Santer, R.D. & Hebets, E.A. (2010). Multimodal courtship efficacy of *Schizocosa retrorsa* wolf spiders: implications of an additional signal modality. *Behav. Ecol.*, 21, 701–707.

Rundus, A.S., Sullivan-Beckers, L., Wilgers, D. & Hebets, E.A. (2011). Females are choosy in the dark: environment-dependent reliance on courtship components and its impact on fitness. *Evoution*, 65, 268–282.

Schluter, D. (1995). Adaptive radiation in sticklebacks – trade–offs in feeding performance and growth. *Ecology*, 76, 82–90.

Schluter. D. & McPhail, J.D. (1992). Ecological character displacement and speciation in sticklebacks. *Am. Nat.*, 140, 85–108.

Shamble, P.S., Wilgers, D.J., Swoboda, K.A. & Hebets, E.A. (2009). Courtship effort is a better predictor of mating success than ornamentation for male wolf spiders. *Behav. Ecol.*, 20, 1242–1251.

Stratton, G. (2005). Evolution of ornamentation and courtship behavior in *Schizocosa*: insights from a phylogeny based on morphology (Araneae, Lycosidea). *J. Arachnol.*, 33, 347–376.

Sullivan–Beckers, L. & Cocroft, R.B. (2010). The importance of female choice, male–male competition, and signal transmission as causes of selection on male mating signals. *Evolution*, 64, 3158–3171.

Uetz, G.W., Papke, R. & Kiline, B. (2002). Influence of feeding regime on body size body condition and male secondary sexual character in *Schizocosa ocreata* wolf spiders (Araneae, Lycosidae): Condition-Dependence in a visual signaling trait. *J. Arachnol.*, 30, 461–469.

Vaccaro, R., Uetz, G.W. & Roberts, A.J. (2010). Courtship and mating behavior of the wolf spider *Schizocosa bilineata* (Araneae: Lycosidae). *J. Arachnol.*, 38, 452–459.

Walker, T.J. (1957). Specificity in the response of female tree crickets (Orthoptera, Gryllidae, Oecanthinae) to calling songs of the males. *Ann. Ent. Soc. Am.*, 50, 626–636.

Wollerman, L. (1998). Stabilizing and directional preferences of female *Hyla ebraccata* for calls differing in static properties. *Anim. Behav.*, 55, 1619–1630.
