# Supplementary material for: Diversification under sexual selection: the relative roles of mate preference strength and the degree of divergence in mate preferences
Source: Ecol Lett. 2013 Jul 1;16(8):964–74. doi: 10.1111/ele.12142 (PMC3757319; doi:10.1111/ele.12142)
Supplement: Supplementary file 3 [file ele0016-0964-SD3.doc]

**Appendix S3 *Consequences of using different criteria for defining a peak preference in linear mate preference functions that do not plateau***

With linear or open mate preference functions that reach a plateau, investment beyond a certain point does not increase attractiveness. Further, even if the preference function does not reach a plateau, there may nevertheless be diminishing returns if the cost of developing even more extreme mating displays outweighs the increase in attractiveness that is achieved. Consequently, we defined preference peaks even in case studies (*Gasterosteus* and *Schizocosa*; see Appendix S1) where preferences were open and we used linear regression to describe them. We defined the peak at 75% response. Here we show that this choice does not affect our analyses: using higher peaks simply make the ∆t~∆p relationship shallower (Table S1; Fig. S1).

**Table S1** Effect of different criteria for defining peak preference in linear preferences lacking a plateau, illustrated with the *Schizocosa* case study. Note that the ∆p term becomes slightly weaker (lower *F*–ratio, higher *P*–value) as the thresholds go from 75% to 95%

| criteria for peak preference | term | *F* | df | *P* |
| --- | --- | --- | --- | --- |
| 75% peaks | ∆p | 7.75 | 1,13 | **0.016** |
|  | pref. strength | 3.17 | 1,13 | 0.098 |
|  | ∆p × pref. strength | 5.53 | 1,13 | **0.035** |
|  |  |  |  |  |
| 85% peaks | ∆p | 6.33 | 1,13 | **0.026** |
|  | pref. strength | 2.44 | 1,13 | 0.14 |
|  | ∆p × pref. strength | 4.66 | 1,13 | **0.05** |
|  |  |  |  |  |
| 95% peaks | ∆p | 5.58 | 1,13 | **0.034** |
|  | pref. strength | 2.09 | 1,13 | 0.17 |
|  | ∆p × pref. strength | 4.25 | 1,13 | **0.06** |


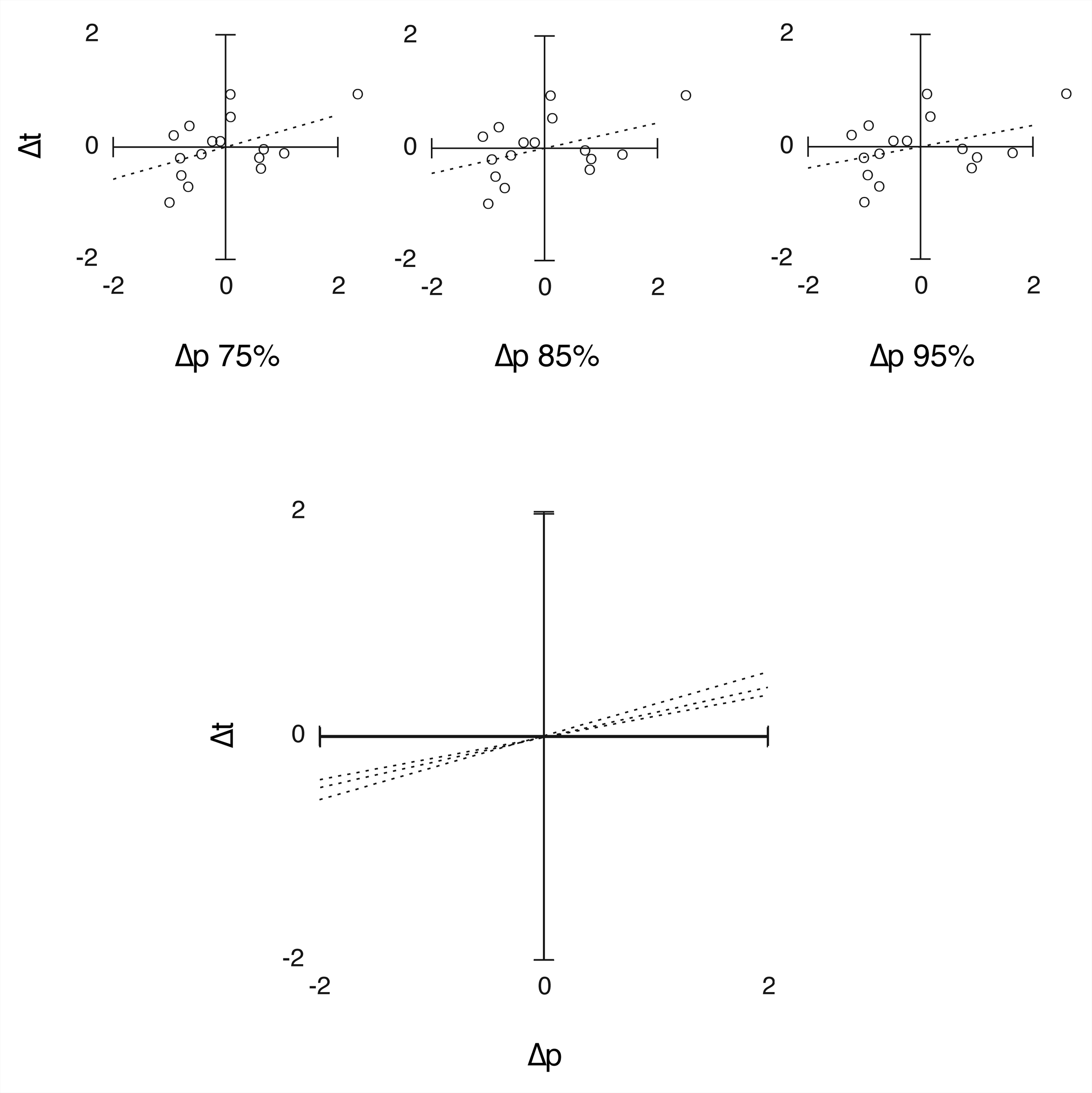


**Figure S1** Effect of different criteria for defining peak preference in linear preferences lacking a plateau, illustrated with the *Schizocosa* case study. Top row: from left to right, the panels show the ∆t~∆p relationship for 75%, 85% and 95% female response–level thresholds for defining peak preference. The bottom panel compares the three regression lines, showing the corresponding change in slope, which became slightly shallower with increasing thresholds.
